# Supplementary material for: The Small RNA Universe of Capitella teleta
Source: Front Mol Biosci. 2022 Feb 25;9:802814. doi: 10.3389/fmolb.2022.802814 (PMC8915122; doi:10.3389/fmolb.2022.802814)
Supplement: Supplementary file 1 [file DataSheet1.ZIP › Supplement/known/CAPTEscaffold_192_13673.pdf]

Provisional ID : CAPTEscaffold_192_13673
Score total : 4.8
Score for star read(s) : -1.3
Score for read counts : 0
Score for mfe : 1.5
Score for randfold : 1.6
Score for cons. seed : 3
Total read count : 9538
Mature read count : 8354
Loop read count : 1
Star read count : 1183

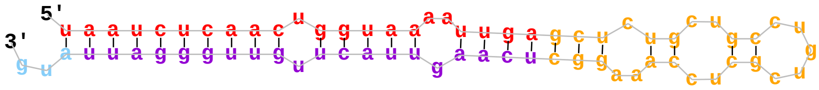

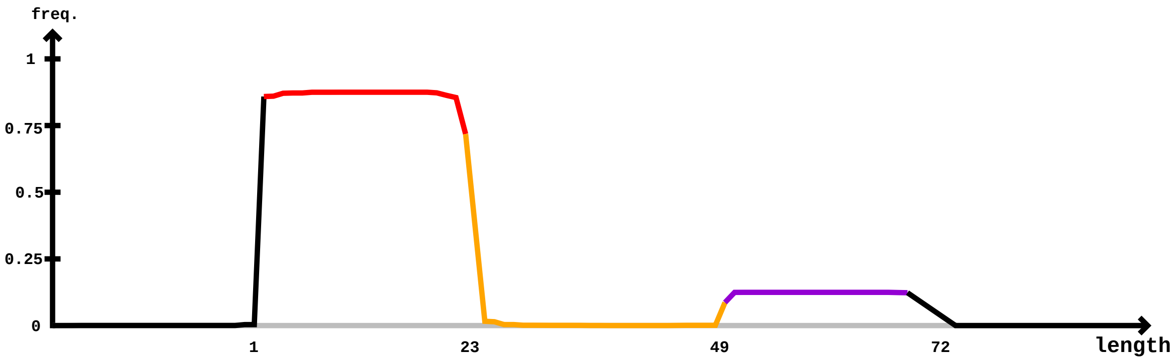

Mature Star

```
5'- gccauuuugugauagucgucuaaucucaacugguaaaauugagcucugcugccugucgcuccaaaggcucaaguuacuuguugggauuaugcgaagucucauaacuaaccca  -3'  obs
    gccauuuugugauagucgucuaaucucaacugguaaaauugagcucugcugccugucgcuccaaaggcucaaguuacuuguugggauuaugcgaagucucauaacuaaccca        exp
    gccauuuugugauagucgucuaaucucaacugguaaaauugagcucugcugccugucgcuccaaaggcucaaguuacuuguugggauuaugcgaagucucauaacuaaccca        known
    ......(((((...((((.((((((((((.(((((..(((((((.((..((.....))..))..))))))).))))).))))))))))).))))....))))))........   reads   mm   sample

    gccauuuugugauagucguc............................................................................................   1   0   seq
    .ccauuuugugauagucguc............................................................................................   2   0   seq
    ..................ucuaaucucaacugguaaaau.........................................................................   12   0   seq
    ..................Acuaaucucaacugguaaaau.........................................................................   1   1   seq
    ..................ucuaaucucaacugguaaaauu........................................................................   14   0   seq
    ..................ucuaaucucaacugguaaaauug.......................................................................   4   0   seq
    ...................cuaaucucaacugguaaaauug.......................................................................   1   0   seq
    ....................uaaucuUaacugguaaaa..........................................................................   1   1   seq
    ....................uaaucucaacugguaaaa..........................................................................   22   0   seq
    ....................Naaucucaacugguaaaau.........................................................................   1   1   seq
    ....................uaaucucaacugguaaaau.........................................................................   75   0   seq
    ....................uaaucucaGcugguaaaauu........................................................................   1   1   seq
    ....................uaaucucaaAugguaaaauu........................................................................   1   1   seq
    ....................Gaaucucaacugguaaaauu........................................................................   1   1   seq
    ....................uaaucucaacugguaaaauu........................................................................   59   0   seq
    ....................uaaucucaacugguaaaauC........................................................................   3   1   seq
    ....................uaaucucaaAugguaaaauug.......................................................................   1   1   seq
    ....................uGaucucaacugguaaaauug.......................................................................   1   1   seq
    ....................uaaucuAaacugguaaaauug.......................................................................   2   1   seq
    ....................Aaaucucaacugguaaaauug.......................................................................   6   1   seq
    ....................uaaAcucaacugguaaaauug.......................................................................   1   1   seq
    ....................uaaucucaacugguaaaaAug.......................................................................   1   1   seq
    ....................uaaucucaacAgguaaaauug.......................................................................   3   1   seq
    ....................Gaaucucaacugguaaaauug.......................................................................   1   1   seq
    ....................uaaucucaacugguaaaauug.......................................................................   1280   0   seq
    ....................uaaucucaGcugguaaaauug.......................................................................   1   1   seq
    ....................uaaucucaacuAguaaaauug.......................................................................   3   1   seq
    ....................uaaucucaUcugguaaaauug.......................................................................   1   1   seq
    ....................uaaAcucaacugguaaaauuga......................................................................   8   1   seq
    ....................uGaucucaacugguaaaauuga......................................................................   2   1   seq
    ....................Naaucucaacugguaaaauuga......................................................................   2   1   seq
```

Mature Star

```
gccauuuugugauagucgucuaaucucaacugguaaaauugagcucugcugccugucgcuccaaaggcucaaguuacuuguugggauuaugcgaagucucauaacuaaccca
....................uaaucucaacugguaUaauuga......................................................................  2     1  seq
....................uaaucucaacugguaaaaCuga......................................................................  1     1  seq
....................uaaucucaacugguaaaauuAa......................................................................  4     1  seq
....................uaaucucaacugguaaaauuNa......................................................................  1     1  seq
....................uaaucucaacugguGaaauuga......................................................................  5     1  seq
....................uaaucucaacCgguaaaauuga......................................................................  1     1  seq
....................uaaucucaacugguaaaauuga......................................................................  6300  0  seq
....................uaaucucaacugCuaaaauuga......................................................................  1     1  seq
....................uaauGucaacugguaaaauuga......................................................................  1     1  seq
....................uaaucucaacuUguaaaauuga......................................................................  3     1  seq
....................uaaucucaacugguaaaauugU......................................................................  7     1  seq
....................uaaucucaGcugguaaaauuga......................................................................  3     1  seq
....................uaaucucaacugguaaaaAuga......................................................................  1     1  seq
....................uaaucucaacugUuaaaauuga......................................................................  1     1  seq
....................uaaucuUaacugguaaaauuga......................................................................  5     1  seq
....................uaaucucaacugguaaaauuCa......................................................................  1     1  seq
....................uaaucucaacuAguaaaauuga......................................................................  6     1  seq
....................uaaucucaacuggCaaaauuga......................................................................  1     1  seq
....................Aaaucucaacugguaaaauuga......................................................................  21    1  seq
....................uaaucAcaacugguaaaauuga......................................................................  3     1  seq
....................uaaucucaacugguaaaauugG......................................................................  18    1  seq
....................Caaucucaacugguaaaauuga......................................................................  5     1  seq
....................uaaucuAaacugguaaaauuga......................................................................  2     1  seq
....................uaaucucaacuCguaaaauuga......................................................................  2     1  seq
....................uaaucucaacugguaaUauuga......................................................................  1     1  seq
....................uaaucucaaAugguaaaauuga......................................................................  9     1  seq
....................uaaucucaUcugguaaaauuga......................................................................  2     1  seq
....................uaaucucGacugguaaaauuga......................................................................  3     1  seq
....................Gaaucucaacugguaaaauuga......................................................................  4     1  seq
....................uaaucucaacugguaaaauCga......................................................................  1     1  seq
....................uaaucucGacugguaaaauugag.....................................................................  54    1  seq
....................uaaucucaacugguaaaauugaA.....................................................................  34    1  seq
....................uaaucucaacugguaaaauAgag.....................................................................  1     1  seq
....................uaaucucaacugguaaaauugag.....................................................................  189   0  seq
....................uaaucucaacugguaaaauugagA....................................................................  7     1  seq
.....................aaucucaacugguaaaauug.......................................................................  1     0  seq
.....................aaucucaacugguaaaauuga......................................................................  7     0  seq
.....................Uaucucaacugguaaaauuga......................................................................  1     1  seq
.....................aaucucaacugguaaaauugagc....................................................................  5     0  seq
......................aucucaacugguaaaauugagc....................................................................  4     0  seq
......................aucucaacugguaaaauugagcu...................................................................  97    0  seq
......................aucucaacugguaaaauugagcA...................................................................  1     1  seq
......................auAucaacugguaaaauugagcu...................................................................  1     1  seq
......................aucucaacugguaaaauAgagcu...................................................................  1     1  seq
.......................ucucaacugguaaaauugagc....................................................................  1     0  seq
.......................ucucaacugguaaaauugagcu...................................................................  2     0  seq
.......................ucucaacugguaaaauugagcucu.................................................................  1     0  seq
.......................ucucaacugguaaaauugagcucugcugcc...........................................................  1     0  seq
.......................ucucaacugguaaaauugagcucugcugccu..........................................................  4     0  seq
.........................ucaacugguaaaauugagcucu.................................................................  23    0  seq
.........................ucaacugguaaaauugagcucugU...............................................................  1     1  seq
.........................ucaacugguaaaauugagcucugUu..............................................................  1     1  seq
.........................ucaacugguaaaauugagcucugcugccugu........................................................  2     0  seq
............................acugguaaaauugagcucugcu..............................................................  1     0  seq
...........................................cucugcugccugucgcuccaaaggcu...........................................  1     0  seq
...............................................................aaggcucaaguuacuuguuggga..........................  4     0  seq
................................................................aggcucaaguuacuuguuggga..........................  4     0  seq
....................................................................ucaaguuacuuguugggau.........................  6     0  seq
....................................................................Gcaaguuacuuguugggauu........................  1     1  seq
....................................................................ucaaguuacuAguugggauu........................  1     1  seq
....................................................................uUaaguuacuuguugggauu........................  1     1  seq
....................................................................ucaaguuacuuguuAggauu........................  1     1  seq
....................................................................ucaaguuacuuguugCgauu........................  1     1  seq
....................................................................Ncaaguuacuuguugggauu........................  1     1  seq
....................................................................ucaaguuacuuguugggauu........................  494   0  seq
....................................................................Acaaguuacuuguugggauu........................  2     1  seq
....................................................................ucaagAuacuuguugggauu........................  1     1  seq
....................................................................ucaaguuacuuguugAgauu........................  1     1  seq
....................................................................ucaaguuaAuuguugggauua.......................  1     1  seq
....................................................................Acaaguuacuuguugggauua.......................  1     1  seq
```

```
                    Mature                                          Star
gccauuuugugauagucgucuaaucucaacugguaaaauugagcucugcugccugucgcuccaaaggcucaaguuacuuguugggauuaugcgaagucucauaacuaaccca
....................................................................uUaaguuacuuguugggauua.......................   2     1     seq
....................................................................ucaaguuacuugAugggauua.......................   1     1     seq
....................................................................ucaaguuacuuguugggauua.......................   174   0     seq
....................................................................ucaaguuacuuguugAgauua.......................   1     1     seq
....................................................................ucaaguuacuuguugggauuU.......................   2     1     seq
....................................................................ucaaguuacuuguuAggauua.......................   1     1     seq
....................................................................ucaaguuacuuAuugggauuau......................   1     1     seq
....................................................................ucaaguuacuuguugggauuau......................   100   0     seq
....................................................................ucaaguuacuuguugggauuaA......................   5     1     seq
....................................................................ucaaguuacuuguugggauuGug.....................   1     1     seq
....................................................................ucaaguuacuuguugggauuauA.....................   4     1     seq
....................................................................ucaaguuacuuguugggauuaug.....................   10    0     seq
....................................................................ucaaguuacuuguugggauuauU.....................   4     1     seq
.....................................................................caaguuacuuguugggauu........................   11    0     seq
.....................................................................caaguuacuuguugggauua.......................   1     0     seq
.....................................................................caaguuacuuguugggauuau......................   126   0     seq
.....................................................................Uaaguuacuuguugggauuau......................   1     1     seq
.....................................................................caaguuacuuguugggauuaA......................   1     1     seq
.....................................................................cUaguuacuuguugggauuau......................   1     1     seq
.....................................................................caaguuacuuguugggauuaug.....................   191   0     seq
.....................................................................caaguuacuuguugggauuauA.....................   1     1     seq
.....................................................................caaguuacuuguugggauuauU.....................   24    1     seq
.....................................................................caaguuacuuguugggauuaugU....................   6     1     seq
......................................................................aaguuacuuguugggauuaug.....................   1     0     seq
......................................................................aaguuacuuguugggauuaugc....................   1     0     seq
```
